# Automated detection of lung nodules and coronary artery calcium using artificial intelligence on low-dose CT scans for lung cancer screening: accuracy and prognostic value

**DOI:** 10.1186/s12916-021-01928-3

**Published:** 2021-03-04

**Authors:** Jordan Chamberlin, Madison R. Kocher, Jeffrey Waltz, Madalyn Snoddy, Natalie F. C. Stringer, Joseph Stephenson, Pooyan Sahbaee, Puneet Sharma, Saikiran Rapaka, U. Joseph Schoepf, Andres F. Abadia, Jonathan Sperl, Phillip Hoelzer, Megan Mercer, Nayana Somayaji, Gilberto Aquino, Jeremy R. Burt

**Affiliations:** 1 Department of Radiology, Medical University of South Carolina, Charleston, SC 29403 USA; 2 Siemens Healthineers, Princeton, NJ USA; 3 MUSC-ART, Cardiothoracic Imaging, 25 Courtenay Drive, MSC 226, 2nd Floor, Rm 2256, Charleston, SC 29425 USA

**Keywords:** Convolutional neural networks, Deep learning, Artificial intelligence, Lung cancer screening, Coronary artery disease, Cardiothoracic imaging

## Abstract

**Background:**

Artificial intelligence (AI) in diagnostic radiology is undergoing rapid development. Its potential utility to improve diagnostic performance for cardiopulmonary events is widely recognized, but the accuracy and precision have yet to be demonstrated in the context of current screening modalities. Here, we present findings on the performance of an AI convolutional neural network (CNN) prototype (AI-RAD Companion, Siemens Healthineers) that automatically detects pulmonary nodules and quantifies coronary artery calcium volume (CACV) on low-dose chest CT (LDCT), and compare results to expert radiologists. We also correlate AI findings with adverse cardiopulmonary outcomes in a retrospective cohort of 117 patients who underwent LDCT.

**Methods:**

A total of 117 patients were enrolled in this study. Two CNNs were used to identify lung nodules and CACV on LDCT scans. All subjects were used for lung nodule analysis, and 96 subjects met the criteria for coronary artery calcium volume analysis. Interobserver concordance was measured using ICC and Cohen’s kappa. Multivariate logistic regression and partial least squares regression were used for outcomes analysis.

**Results:**

Agreement of the AI findings with experts was excellent (CACV ICC = 0.904, lung nodules Cohen’s kappa = 0.846) with high sensitivity and specificity (CACV: sensitivity = .929, specificity = .960; lung nodules: sensitivity = 1, specificity = 0.708). The AI findings improved the prediction of major cardiopulmonary outcomes at 1-year follow-up including major adverse cardiac events and lung cancer (AUC_MACE_ = 0.911, AUC_Lung Cancer_ = 0.942).

**Conclusion:**

We conclude the AI prototype rapidly and accurately identifies significant risk factors for cardiopulmonary disease on standard screening low-dose chest CT. This information can be used to improve diagnostic ability, facilitate intervention, improve morbidity and mortality, and decrease healthcare costs. There is also potential application in countries with limited numbers of cardiothoracic radiologists.

**Supplementary Information:**

The online version contains supplementary material available at 10.1186/s12916-021-01928-3.

## Background

Atherosclerotic cardiovascular disease (CVD) and lung cancer are the leading causes of death in the USA with CVD leading to overall mortality in adults and lung cancer causing 25% of all cancer deaths [1, 2]. Effective screening and early detection are instrumental in reducing morbidity and mortality, as lung cancer can be diagnosed earlier and therapy can be initiated for cardiovascular disease before symptoms manifest.

Low-dose computed tomography (LDCT) imaging is a well-validated screening tool for lung cancer that significantly reduces mortality [3–7]. Patients receiving LDCT scans typically have major risk factors that predispose them to coronary artery disease and would be highly advantageous to concurrently screen for both lung cancer and assess coronary calcification burden, which is a well-known marker for subsequent major cardiovascular adverse events [8–10]. Multiple prior studies have shown LDCT to be a feasible tool in estimating coronary artery calcium volume (CACV) using manual or semi-manual techniques [11–14].

Recently developed artificial intelligence (AI) deep learning methods using convolutional neural networks (CNN) have been used for the detection of lung nodules, which has been shown to improve detection sensitivity and reduce reading times [15–17]. Automatic calcium scoring methods, particularly on non-contrast chest CT scans, can introduce large margins of error due to motion and calcium location miscategorization; however, newer techniques could compensate for these limitations.

The purpose of this study was to investigate the performance of a fully automated AI convolutional neural network (CNN, a multi-layered machine learning algorithm which utilizes multiple hidden layers and sequential output patterns that excel at image) in simultaneously detecting solid pulmonary nodules and quantifying CACV on routine LDCT scans of the chest when compared against expert radiologists. In addition, the AI CNN results were evaluated for patient outcomes after at least a 12-month follow-up to evaluate for prognostic value.

## Methods

This retrospective study was approved by the Medical University of South Carolina’s institutional review board with a waiver of informed consent and was conducted in compliance with the Health Insurance Portability and Accountability Act.

### Study population

We evaluated LDCT studies at random that were performed at our institution for patients who underwent routine lung cancer screening between January 2018 and July 2019. The exclusion criteria included age < 18 years old and rejection of the chest CT by AI-RAD due to incompatible image parameters (i.e., CT slice thickness > 3 mm, poor image quality). Standard low-dose lung cancer screening inclusion criteria were utilized [6]. All 117 subjects were used for lung nodule analysis, and 96 subjects met the CT quality criteria for successful CACV segmentation, concordance, and outcomes analysis.

For each patient, demographics, including age, sex, and smoking history, were obtained via chart review. Clinical history including variables such as hypertension, hyperlipidemia, and diabetes, as well as clinical outcomes including major adverse cardiac events, death, hospitalization, and stroke; lung cancer diagnosis; and pulmonary hospitalization, were also documented. Major adverse cardiac event (MACE) was defined as acute coronary syndrome/myocardial infarction hospitalization, percutaneous coronary intervention, or surgical intervention.

### Image acquisition

Acquisitions were performed using one of four Siemens scanners: go.Top, Definition AS+, Flash, and Force. Similar scanning parameters were used for each of the different scanners following the American College of Radiology-Society of Thoracic Radiology (ACR-STR) Practice Parameters [18]. For example, acquisitions using the third-generation dual-source CT system (SOMATOM Force; Siemens, Forchheim, Germany) were performed from the lung apices through the bases, without contrast during breath-hold at end-inspiration. Acquisition parameters included the following: 110 kVp tube-voltage, 40 eff mAs (which was changed to 120 kVp and 70 eff mAs for patients with body mass index of > 30), 192 × 0.6 mm collimation, gantry rotation time of 0.5 s, pitch of 0.7, and effective slice thickness of 1 mm. Images were reconstructed with both soft body and sharp body kernels at an axial slice thickness of 1 mm, according to the standard ACR-STR LDCT guidelines.

### Description of AI neural network process: coronary artery calcium detection

The chest CT calcium detection model was first trained on native, ECG-gated calcium scoring scans with corresponding radiologist-verified ground truth labels for the coronary calcifications. The trained model was then refined on a set of non-contrast-enhanced chest CT scans to obtain the final model. The validation data consisted of 1261 ECG-gated calcium scoring scans and 579 chest CT scans from multiple centers across the USA, Europe, and Asia.

Since the size of the heart can vary substantially from patient to patient, the model computations were performed in a patient-specific scaled coordinate system, in which the heart was scaled to have a consistent size. The training data for the model was used to construct a likelihood model representing a probability that a given coordinate belonged to one of the coronary arteries in the patient-specific coordinate system.

**Fig. 1:**
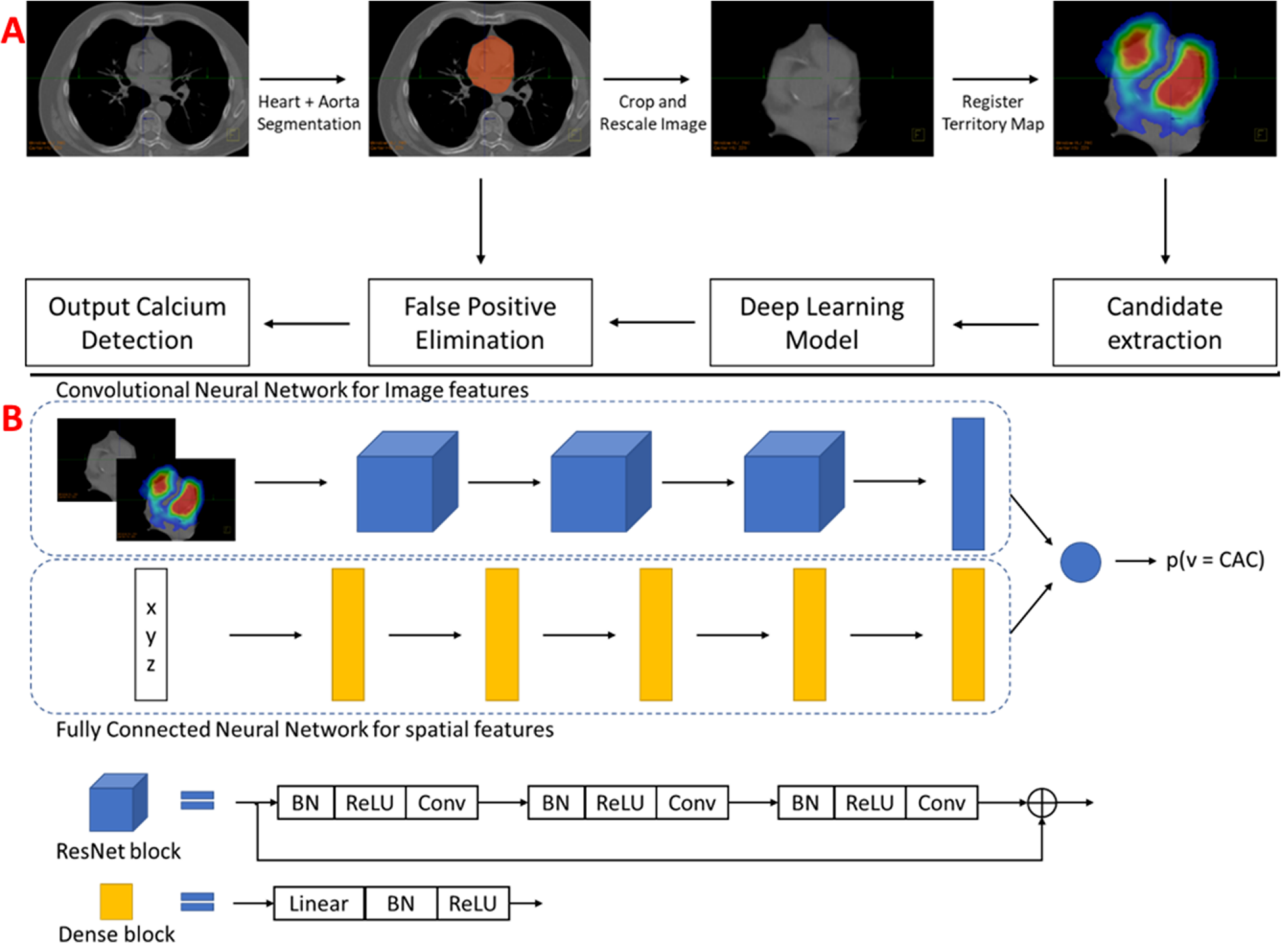
**a** Computational pipeline used for the chest CT calcium detection model. **b** Architecture of the deep learning chest CT calcium detection model used for predicting the probability that each candidate voxel belongs to the coronary arteries. CAC, coronary artery calcium; BN, batch normalization; ReLU, rectified linear unit; Conv, convolutional layer

**Fig. 2:**
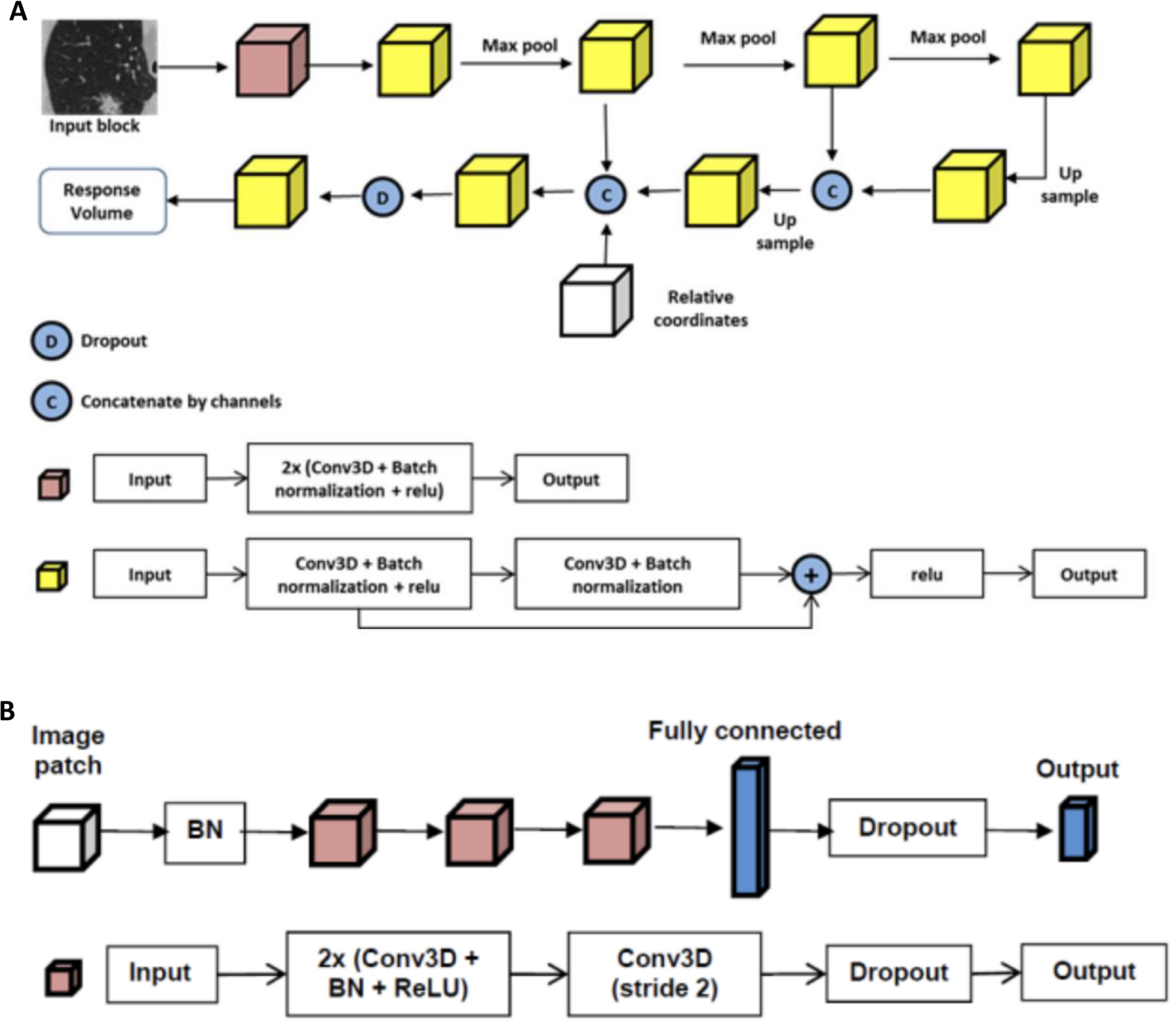
Pictorial representation of the lung nodule detection neural network used for lung nodule detection (**a**) and lung lobe segmentation (**b**). BN, batch normalization; ReLU, rectified linear unit; Conv, convolutional layer

The computational pipeline used for the chest CT calcium detection model is shown in Fig. 1. During model preprocessing, a heart segmentation model (U-Net architecture, trained and validated using 660 chest CT scans) was used to identify and crop the region of interest surrounding the heart. Subsequently, candidate voxels were identified in the cardiac region by thresholding at 130 HU. For each candidate voxel, a small image patch (32 × 32 pixels in axial plane) surrounding it along with the corresponding prior likelihood map was used as image features, and the spatial coordinates of the point in the patient-specific coordinate system were used as additional features. The final neural network model architecture is shown in Fig. 2.

The image features were processed through a convolutional neural network with a ResNet architecture, and the spatial features were processed using a fully connected deep neural network. The outputs from these two models were concatenated and used in the final layer to predict the probability of each candidate voxel being coronary calcifications. In the final stage, an aorta segmentation model was used to remove any false-positive aortic calcifications which might have been mispredicted by the calcium detection model, to obtain the final output from the model.

### Description of AI neural network process: lung nodule detection

**Fig. 3:**
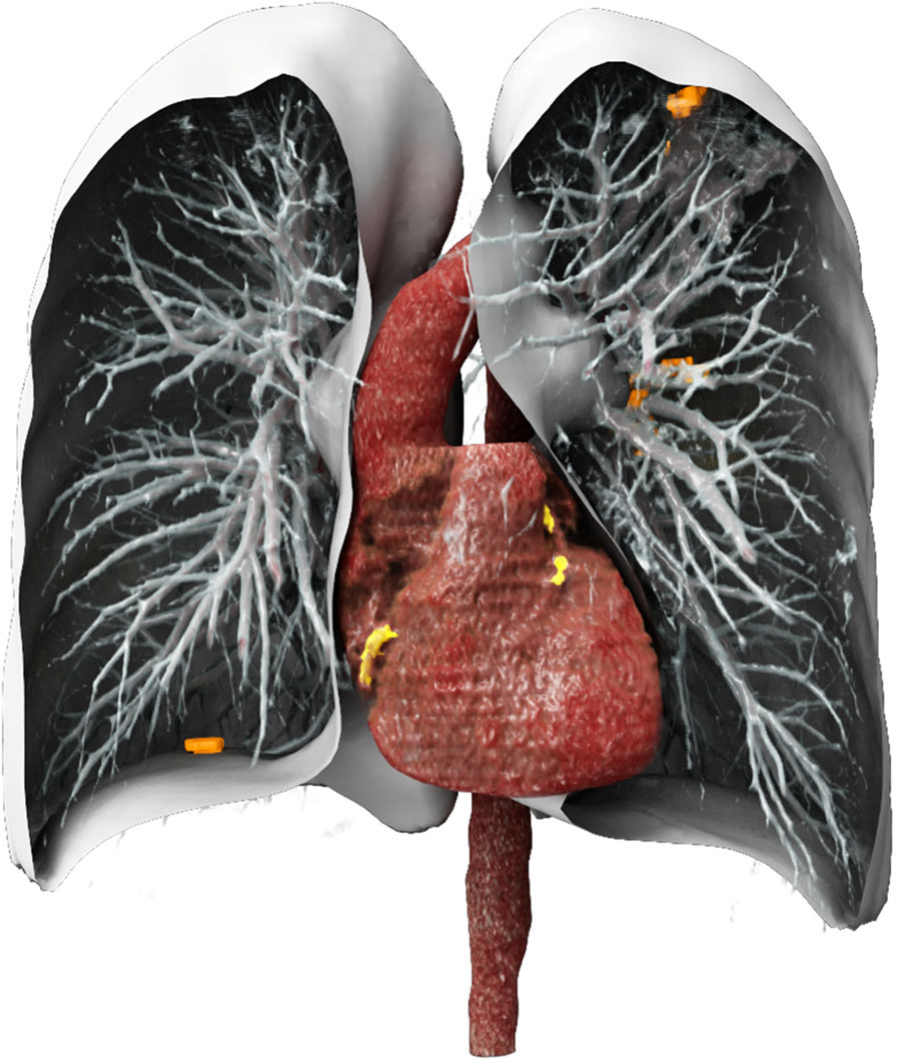
Cinematic volume rendering of the detected coronary calcium (yellow) and lung nodules (orange) along with the lungs, pulmonary vessel, aorta, and heart segmentations from a representative low-dose chest computed tomography

The lung nodule detection model has both lung nodule detection and lung lobe segmentation (nodule localization) capability. Lung nodule detection was performed in a two-step approach including nodule candidate generation (NCG) and false-positive reduction (FPR). The NCG comprises a proposed 3D region network that outputs a few suspicious lesions called “nodule candidates,” for which probability scores were assigned [19]. Each nodule candidate and a small sample of voxels around it were sent to the FPR module, which further assessed the likelihood of the nodule candidate to be a true or false positive via updating the scores generated by the NCG module [20]. Weighted sums of the scores generated by both modules were used to produce the final decision (Fig. 3). The lung nodules were then segmented by an algorithm based on region growing.

AI-RAD also performed lung lobe segmentation for nodule localization. Segmentation masks of the five lung lobes for a given CT chest dataset were computed, which inputs the entire 3D CT volume and outputs probability maps that indicate the likelihood of a voxel belonging to each lobe. A deep image-to-image network in a symmetric convolutional encoder-decoder architecture was utilized [21]. This AI model for lung nodule detection, localization, and segmentation was previously trained on 5000 manually curated chest CT scans and validated against 129 separate CT datasets [22].

### AI RAD companion and measurements

**Fig. 4:**
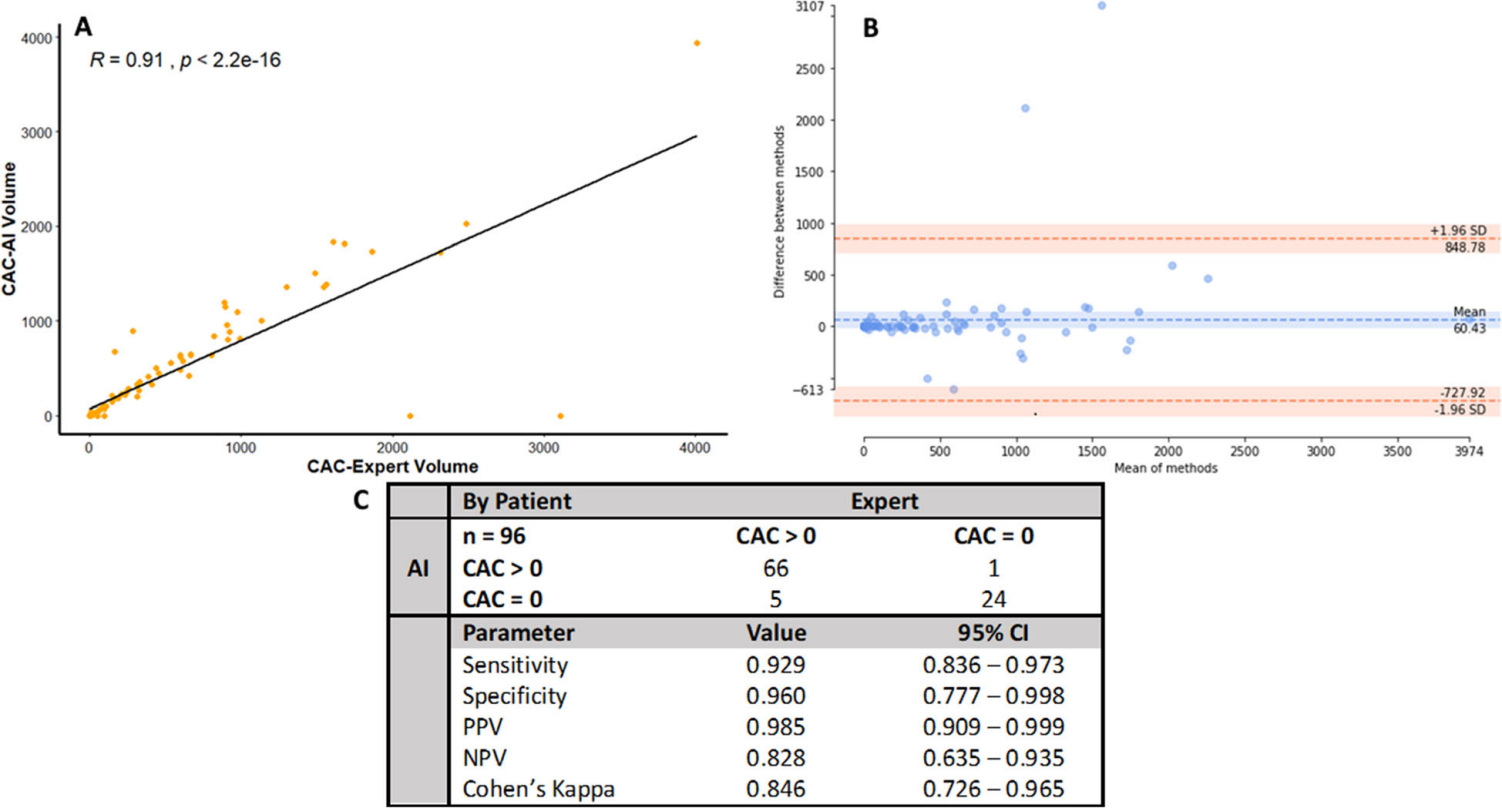
Concordance statistics for AI determination of CACV. **a** Correlation of expert and AI-calculated CACV volumes by Spearman’s method. *R*^2^ = 0.792. Two-tailed ICC = 0.904 (95% CI 0.857–0.936), *p* = < 0.0001. *α* = 0.05. The AI and expert CACV volumes have an excellent agreement that reliably follows a generalized linear trend with few outliers. **b** Bland-Altman plot of the quantitative differences between AI and expert measurements of CACV volume. AI and expert have a mean volume difference of 65.96 for the agreement of CACV volume. The CACV-AI software accurately predicts the CACV volume within acceptable limits compared to the expert. **c** Concordance of expert and AI reads of CACV binned into CACV > 0 and CACV = 0 groups, representing patients with and without CACV, respectively. Expert and AI excellently agree (Cohen’s kappa = 0.846, 95% CI 0.726–0.965) about CACV status with excellent screening parameters (sensitivity = 0.929, specificity = 0.960). CAC-AI volume, coronary artery calcium-artificial intelligence volume; CAC-Expert volume, coronary artery calcium-expert volume; SD, standard deviation; PPV, positive predictive value; NPV, negative predictive value; CI, confidence interval

All LDCT images were evaluated using an ensemble of the previously described chest CT calcium detection and lung nodule detection models in a prototype version of AI-RAD Companion (VA10A, Siemens Healthineers, Erlangen, Germany) to assess for lung nodules (AI-LN) and CACV (AI-CACV). This prototype version of the software platform provides automatic AI-based multi-organ image analysis, visualization, and quantification [22, 23]. AI-LN was asked to report the location and largest 2D diameter of the five largest nodules present as well as classify each patient into two groups: nodules present or nodules absent. The final model output (detected calcium and lung nodules) along with the segmentations of the lungs, the aorta, and the heart is rendered using a cinematic rendering model in Fig. 4.

### Coronary artery calcium volume validation

Manual (semi-automatic) CACV scoring was performed by an expert radiologist on the axial 1.5-mm soft tissue (*B* < 60) reformatted images using TeraRecon (Durham, NC).

### Lung nodule validation

Lung nodule validation was performed on a per-patient and per-nodule basis with two expert radiologist consensus confirmation as the gold standard. The expert radiologists validated AI-LN’s reported nodules and assessed whether each nodule was a true positive (TP), false positive (FP), true negative (TN), or false negative (FN). TN cases were defined as AI-LN reports no nodules and expert radiologists confirmed the lack of any nodules. FN cases were defined as AI-LN reports no nodules, but expert radiologist blinded over-read indicated a nodule was present. A FP nodule was defined as a nodule that was reported by AI-LN but not by expert radiologists. A TP nodule was defined as a nodule that was reported by both AI-LN and expert radiologists to represent a nodule. False-positive nodules were identified, and their true anatomical identity (i.e., osteophyte, atelectasis) was collected for a false-positive analysis.

The AI-RAD lung nodule detection parameters can be adjusted based on the need of the end user. The parameters for this study were as follows: detect up to 30 lung nodules in each chest CT, only detect nodules > 6 mm in the greatest dimension, and report only the largest 5 nodules by the greatest dimension.

Per-patient validation was performed by the presence of false positives and true negatives. Any patient with no nodules determined by an expert radiologist panel and AI-LN was considered to be a true-negative patient. Any patient with one or more false positives was listed as false positive for the purpose of per-patient validation. Only patients with no false positives were listed as a true-positive patient for the purpose of concordance.

### Determining prognostic value

First, univariate statistics of clinical demographics and risk factors were performed to assess for possible significant predictors and confounding variables among all 117 patients. Variables included sex, race, current smoking status, diagnosis of diabetes, hypertension, hyperlipidemia, tuberculosis exposure, asbestos exposure, family history of lung cancer, chronic obstructive pulmonary disease, interstitial lung disease, history of cardiac disease, family history of cardiac disease, stroke/transient ischemic attack, daily aspirin use, and AI and Expert CAC Score. Simple logistic regressions were then performed comparing the accuracy of AI-RAD and expert radiologist reads of CACV and lung nodules for the prediction of cardiopulmonary outcomes. Comparisons of AI-RAD and expert radiologist reads for negative outcome predictions were then performed using ROC curves and log-likelihood test for the logistic models. Individual outcomes were then analyzed by multivariate partial least-squares regression. Correlation biplots and ROC curves were calculated to assess for both strength of correlation and accuracy in the model. Evaluation of the model fit was performed using both *R*^2^ and root mean square error (RMSE). The individual strength of predictors was evaluated using correlation and variable importance in projection (VIP) with the inclusion criteria being VIP > 1 and 95% confidence interval significant from zero.

### Root analysis of false-positive nodules

Initially, univariate statistics were performed to assess for confounding variables associated with having at least one FP nodule per patient. Significant variables were then analyzed by simple logistic regression for the prediction of FP nodules. Log-likelihood tests were performed to assess for the significance of the model compared to a null hypothesis of no impact on the predictor. Logistic regression probability curves were then generated for any significant models to assess for quantitative impact of the predictor on the detection of any FP patient. Additionally, each individual FP nodule was assessed by an expert radiologist post hoc to qualitatively identify the true anatomical identity of each nodule. Qualitative results were then reported in tabular format.

### Statistical analysis

Demographics, risk factors, summary statistics, and concordance analysis were calculated using XLSTAT 20.1.2 Addinsoft (2020). (XLSTAT statistical and data analysis solution. New York, USA. https://www.xlstat.com). Continuous variables were assessed for normality by visualization and the Shapiro-Wilk test and visualization by histogram (not reported). Continuous variables were reported as mean and standard deviation if normally distributed and median plus interquartile range if non-normally distributed. Tests for association were assessed with two-tailed *t* tests for continuous normal variables and Mann-Whitney *U* tests for non-normally distributed continuous variables. Categorical measures of association were assessed using Fisher’s exact tests given the small counts of some observations. 95% confidence intervals for Cohen’s kappa and screening parameters were calculated using a normal approximation interval. Intraclass correlation coefficients were reported along with Spearman’s *R* for concordance and reliability of continuous variables. Bland-Altman plots were reported for assessing the quantitative differences between observers for continuous variables (CACV values). Bland-Altman plots were generated using the pyCompare (2018) package for python 3.6 (DOI: 10.5281/zenodo.1256204). Univariate statistics were reported with the same procedure as demographics and risk factors. Bonferroni alpha correction was not applied. Exploratory simple logistic regression and scatterplot visualization were performed in R (v3.6.3). Partial least squares regression was performed in XLSTAT for automatic generation of correlation biplots, ROC curves, and standardization of viewing.

## Results

### Neural network architecture

Figure 1 describes the design of the network utilized for CACV detection. Figure 2 describes the neural network used for lung nodule connection. Figure 3 demonstrates the combined cinematic reconstruction of the heart and lungs simultaneously analyzed by both networks and displaying CACV and lung nodules (see the “Methods” section for detailed components of neural network construction, training, and architecture.

### Study population demographics, clinical attributes, risk factors, and univariate statistics

Demographics of patients evaluated by both the AI algorithm and expert radiologists for automated coronary calcium scoring and lung nodule detection are reported in Additional file 1: Table S1. Comparison of risk factors and clinical attributes between patients with expert radiologist-determined nodules, AI-determined nodules, and patients with CACV > 0 and CACV = 0 is reported in Additional file 1: Table S2. Patients with COPD and a history of cardiac disease were more likely to have CAC (*p* = 0.043, 0.016). Finally, demographics and risk factors associated with both pulmonary outcomes and cardiovascular outcomes were evaluated for the identification of confounding variables (Additional file 1: Tables S3 and S4). There was a total of 11 patients with ACS/MI hospitalization, 11 with PCI/surgical intervention, and 13 with MACE. Twenty-seven patients were hospitalized for pulmonary causes, and 5 patients were diagnosed with lung cancer. A higher CACV by both expert radiologist and AI was significantly associated with all cardiac events (*p* < 0.001).

### Coronary artery calcium volume

Figure 4a describes the correlation between the expert radiologist and AI-RAD measurement of CACV. The two measurements are highly correlated (*R*^2^ = 0.792) and in excellent agreement (two-tailed ICC = 0.904, 95% CI 0.857–0.936). Figure 4b explores the quantitative differences in agreement between the two observers (AI-RAD and expert radiologist), with a mean CACV disagreement of 60.43 mm^3^. Figure 4c describes the ability of AI-RAD to correctly identify patients with no coronary calcium versus those with CACV greater than zero. Expert radiologist and AI-RAD determinations had an excellent agreement with a Cohen’s kappa of 0.846 (95% CI 0.726–0.965). The sensitivity and specificity were 0.929 and 0.960, respectively. The positive predictive value was 0.985. The overall rate of false positives and false negatives was low (9% combined; 6% FN, 3% FP).

**Fig. 5:**
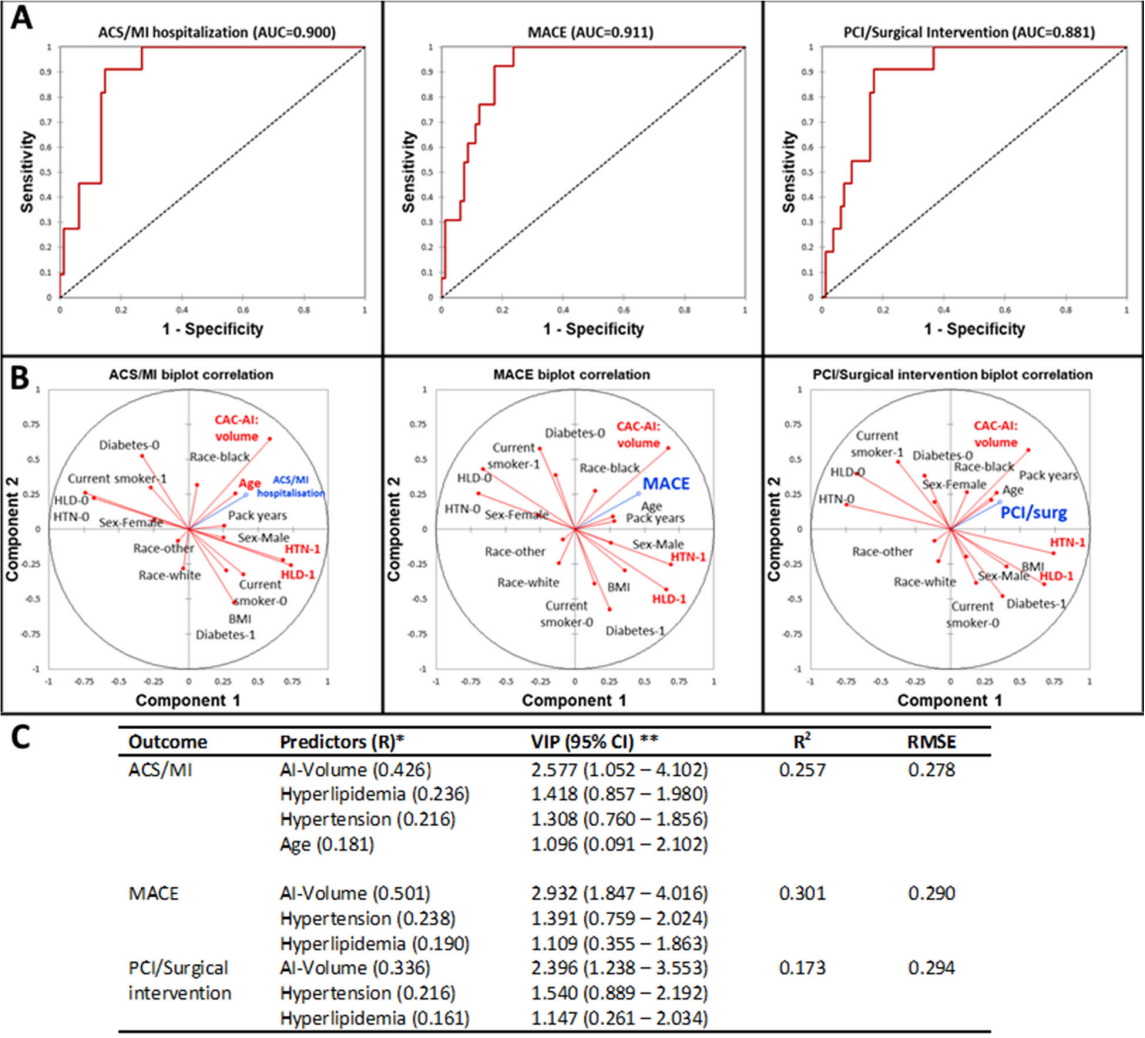
Multivariate cardiovascular outcome modeling using clinical risk factors and CACV-AI volume as predictors in partial least squares regression (PLS). CACV-AI volume was the largest predictor of acute coronary syndrome/myocardial infarction, major adverse cardiac events (MACE), and percutaneous coronary intervention/coronary artery bypass grafting in 1 year of follow-up (AUC = 0.900, 0.911, 0.811, respectively). **a** Receiver-operator curves describing predictive power of individual cardiovascular outcomes. **b** Correlation biplots describing the magnitude and direction of associations of predictors with individual cardiovascular outcomes. **c** Model parameters. Variables with VIP > 1 and 95% CI significant from zero were considered important in the model. The models have a moderate fit and excellently predict cardiovascular events in 1 year of follow-up. ACS/MI, acute coronary syndrome/myocardial infarction; MACE, major adverse cardiac event; PCI, percutaneous coronary intervention; AUC, area under the curve; BMI, body mass index; HTN, hypertension; HLD, hyperlipidemia; VIP, variable importance projection; RMSE, root mean squared error; CI, confidence interval; CAC-AI Volume, coronary artery calcium-artificial intelligence volume; CAC-Expert volume, coronary artery calcium-expert volume

In Fig. 5a, acute coronary syndrome, myocardial infarction hospitalization (ACS/MI hospitalization), MACE, and percutaneous coronary intervention/surgical intervention (coronary artery bypass grafting) were excellently predicted by a combination of AI-RAD and cardiovascular risk factors in all three cases (area under the curve (AUC), 0.900, 0.911, 0.881, respectively). Figure 5b describes the correlation of each predictor with the outcome variable for each cardiovascular endpoint. Variables significant in the PLS model are highlighted in red while the outcomes are highlighted in blue. Hypertension, hyperlipidemia, and AI-RAD were significant in each of the three models (*p* < 0.05). Figure 5c demonstrates the model characteristics for each endpoint. All three endpoint variations are moderately explained by the significant predictors (McFadden *R*^2^ = 0.257 (ACS/MI), 0.301 (MACE), 0.173 (PCI/Surgical intervention) with minimal RMSE (0.278, 0.290, 0.294, respectively)), and all variable importance measured by the PLS-VIP method (VIP > 1, 95% CI > 0). Additional file 1: Table S5 shows the logistic regression model parameters and odds ratios for CACV. Logistic regression models were found to be inferior to PLS regression models in this cohort. Additional file 1: Table S6 compares the PLS model parameters with and without the AI component. Additional file 1: Figures S1 to S3 demonstrate similar AUCs for the prediction of cardiovascular outcomes by expert and AI CACV by simple logistic regression.

### Lung nodules

**Fig. 6:**
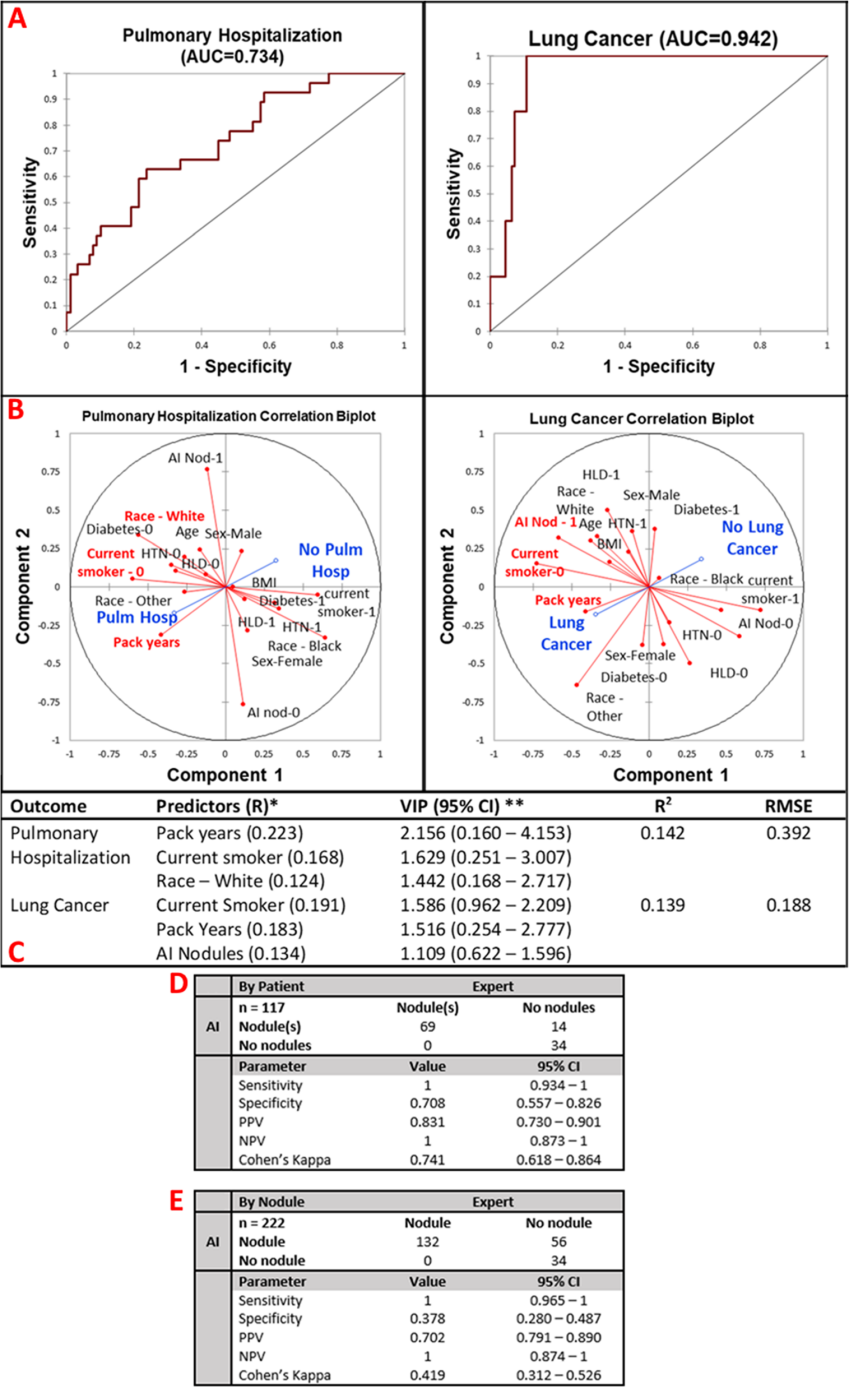
Multivariate pulmonary outcome performance and concordance analysis. AI-detected lung nodules were not a significant predictor of pulmonary hospitalization but were a significant predictor for diagnosis of lung cancer in 1 year of follow-up. **a** ROC curves of prediction of pulmonary hospitalization (AUC = 0.734) and lung cancer (AUC = 0.942). **b** Correlation biplots for pulmonary hospitalization and lung cancer evaluating the magnitude and direction of association of each predictor with the outcome. **c** Model parameters. Pulmonary hospitalization is predicted by pack-years, current smoking status, and White race. *R*^2^ = 0.142, RMSE = 0.392. Lung cancer is significantly predicted by current smoking status, pack-years, and presence of AI-predicted nodules. *R*^2^ = 0.139, RMSE = 0.188. **d** By patient AI analysis of nodules is excellently sensitive and adequately specific for the detection of any lung nodule in a patient (sensitivity = 1, specificity = 0.708). Both AI and expert have a high concordance of diagnosis of lung nodules in a patient (Cohen’s kappa = 0.741). **e** The by-nodule analysis was also highly specific and poorly sensitive with an overall moderate agreement between the expert and AI software (sensitivity = 1, specificity = 0.378, Cohen’s kappa = 0.419). AUC, area under the curve; BMI, body mass index; HTN, hypertension; HLD, hyperlipidemia; VIP, variable importance predictor; RMSE, root mean squared error; CI, confidence interval; PPV, positive predictive value; NPV, negative predictive value

Figure 6a describes the predictive power of these variables for pulmonary hospitalization and lung cancer at 12-month follow-up (AUC = 0.734, 0.942, respectively) by multivariate partial least squares regression. Figure 6b represents the correlation of all predictor variables with the outcomes. Pulmonary hospitalization was correlated with White race, pack-years of smoking, and current smoking status. Lung cancer was correlated with the detection of nodules by the AI, pack-years smoked, and current smoking status. Figure 6c highlights the model parameters. McFadden *R*^2^ was 0.142 and 0.139 for the explanation of pulmonary hospitalization and lung cancer, respectively.

Figure 6d demonstrates the per-patient screening parameters and concordance values for AI-RAD compared to expert radiologists. AI-expert radiologist interobserver variability (Cohen’s kappa) was 0.741 (95% CI 0.618–0.864). The per-patient sensitivity and specificity of AI-RAD were 1 and 0.708, respectively. Fourteen out of 117 patients (12.0%) were identified as having a false-positive nodule. Figure 6e describes the per-nodule concordance and screening parameters for AI-RAD detection of individual lung nodules. The per-nodule analysis was sensitive, detecting every nodule (sensitivity = 1), but poorly specific (specificity = 0.378). There was a total of 56 false-positive nodules identified by the AI software out of a total of 222 nodules (25.2%).

Additional file 1: Table S7 describes the univariate statistics used for the evaluation of false-positive analysis by logistic regression. Age is significantly associated with false-positive nodules (*p* = 0.01). Additional file 1: Figure S4A describes the prediction of false positives by nodules by age, which was a significant variable in univariate analysis (68.7 vs 65.8, *p* = 0.01). Age is a moderate predictor of having a false-positive nodule detected by AI (AUC = 0.666, McFadden *R*^2^ = 0.06, Pr > chi (age) = 0.01, log-likelihood test = 0.007). Additional file 1: Figure S4B describes the probability curve of having at least one false-positive nodule as a function of age. There is roughly a 25% chance of a false-positive nodule at age 64. The mean age in this cohort is 68 years for those with nodules and 64 years for those without nodules. Additional file 1: Figure S4C lists the true anatomical identity of the false-positive nodules. Most false positives were identified as atelectasis, extrapleural fat, infection, and protruding osteophytes from thoracic vertebral bodies. Nine false positives were uncategorizable by the panel of radiologists.

## Discussion

### Value of concurrent automatic detection of lung nodules and coronary calcium

In this study, our goal was to evaluate the accuracy and clinical event predictive power of two simultaneous neural networks when applied to a major screening imaging modality (LDCT). While LDCT screening for lung cancer has been validated across multiple clinical trials, there is also strong evidence that identification of CACV burden reduces mortality in this screening population. The ITALUNG trial screened subjects with 9.3 years of follow-up for cardiovascular mortality as identified by CACV from the LDCT lung cancer screening and found that identification of CACV was associated with a decreased cardiovascular mortality, indicating that at-risk patients were identified and likely treated appropriately [24]. Jacobs et al. in 2010 conclude that CACV can predict all-cause mortality from LDCT screenings [12]. Most recently, a study in 2020 evaluated the impact of significant coronary artery calcification on patient management and concluded that semi-automated CAC detection and quantification directly resulted in a change in management, corroborating the ITALUNG trial findings [25].

### Agreement of AI and expert radiologist determination of coronary calcium volume

In this study, we demonstrate that expert radiologist and AI-RAD measurements are highly correlated (*R*^2^ = 0.792) and in excellent agreement (two-tailed ICC = 0.904, 95% CI 0.857–0.936). The mean quantitative difference was found to be 60.43 mm^3^; however, much of the quantitative differences were found at CACV > 1000 mm^3^, indicating accuracy for most patients and highlighting the need for further work on patients with very large calcium burdens. Another important clinical determination is the absolute presence or absence of coronary calcium. Expert radiologist and AI-RAD determinations had an excellent agreement with a Cohen’s kappa of 0.846 (95% CI 0.726–0.965) for the assignment of patients into these binary categories.

The sensitivity and specificity were 0.929 and 0.960, respectively, and represent a highly accurate and reliable test for this imaging modality. The overall rate of false positives and false negatives was low (9% combined; 6% FN, 3% FP). There were a small number of false-positive reads where the AI-RAD assigned CACV to objects the radiologist omitted. These readings may be explained by several potentially calcified structures in the vicinity of the coronary arteries including the mitral valve annulus, aorta, and pericardium. The lack of contrast used in LDCT further exacerbates these findings as coronary artery segmentation is not possible, an area of improvement needed in the future. We add that false negatives are particularly susceptible to noise reduction features and spatial features that are used to optimize results [26].

While quantitative and binary stratification of CACV is integral for evaluating the concordance of AI-RAD with expert radiologists, recently, a new scoring system (CAC-DRS) has been created that stratifies patients into discrete categories based on the number of coronary vessels involved and the total calcium burden [27]. The CAC-DRS system has recently been validated for risk assessment on non-ECG-gated chest CT images and provides greater risk assessment stratification than standalone CACV. Further study is needed on the ordinal concordance validity of AI-RAD detection of CACV by the CAC-DRS.

### CACV outcomes

As noted above, detection of CAC on LDCT imaging both changes the management of CAD and reduces mortality in at-risk populations. Partial least squares regression was used to predict cardiovascular outcomes as many of the predictors associated with CAD are highly multicollinear and overlap in many patient populations [28, 29]. Acute coronary syndrome/myocardial infarction hospitalization (ACS/MI hospitalization), MACE, and percutaneous coronary intervention/surgical intervention (coronary artery bypass grafting) were excellently predicted by a combination of AI-RAD and cardiovascular risk factors in all three cases (area under the curve (AUC), 0.900, 0.911, 0.881, respectively). Hypertension, hyperlipidemia, and AI-RAD CACV were significant in each of the three models, consistent with the known risk factors of CAD as outlined in Wilson et al. [28]. The addition of AI-CACV improved the model prediction compared to models without AI-CACV. The inclusion of AI-RAD with a few easily obtained cardiovascular risk factors strongly predicts cardiovascular events within 1 year of LDCT imaging, lending credence to the idea that AI-RAD is a robust measurement with the potential to reduce morbidity and mortality.

There were 13 major adverse cardiac events in this study, which is roughly four times more common than 1-year outcomes in published claims data (2.99 events/person-year) [30]. This likely represents selection bias either at a study enrollment or initial LDCT screening level. Our vascular disease frequency (Additional file 1: Table S2) was higher than reported claims data (for example, history of CAD 27.1% vs 16.9%) and likely represents a combination of a sick population and disproportionate enrollment of patients; regional variance and referral bias cannot be excluded. Caution should be exercised when generalizing the outcomes in this data to unrelated populations. Furthermore, the AUCs > 0.9 represent an excellent explanation of cardiovascular outcomes but overpredict compared to the literature (~ 0.7 in the 2016 multi-ethnic study of atherosclerosis); with a low population in the study cohort and differences in the studied populations, generalizability cannot be assumed [31].

### Lung nodule concordance

About 24% of all LDCT scans in the NSLT 2011 trial were read as containing lung nodules, but up to 96% of those nodules were found to be benign. This necessitates AI software to be highly sensitive as the prevalence is high in the pre-test population [32]. We found AI-expert radiologist interobserver variability was excellent (Cohen’s kappa = 0.741 (95% CI 0.618–0.864), and the per-patient sensitivity of AI-RAD was indeed excellent (sensitivity = 1) and moderately specific (specificity = 0.708). These findings indicate that AI-RAD complements the role of LDCT as a screening modality for a high-risk population with a high pre-test probability of lung nodules. Only 14 out of 117 patients (12.0%) were identified as having a false-positive nodule, a finding that closely mimics other well-validated screening modalities such as mammography (11.5% false-positive interpretation rate) [33].

The per-nodule analysis was excellently sensitive, detecting every nodule (sensitivity = 1), but poorly specific (specificity = 0.378). There was a total of 56 false-positive nodules identified by the AI software out of a total of 222 nodules (25.2%). There was 0.48 FP/case for AI-RAD versus 0.33 to 1.39 FP/case reported by a panel of four thoracic imaging experts in the literature [34]. The concordance was similarly only mild in strength due to the rate of false positives (Cohen’s kappa = 0.419). Possible explanations include the lack of contrast generating more false positives for a program trained on mixed imaging modalities or insufficient number of training datasets.

### Lung outcomes

The NLST trial in 2011 concluded that LDCT screening reduced mortality in the screening cohort by identifying cancerous and precancerous lesions and affecting treatment change [4]. Pulmonary hospitalization is adequately predicted by pack-years, current smoking status, and White race (AUC = 0.734, *R*^2^ = 0.142, RMSE = 0.392). The low *R*^2^ and high AUC likely indicate that while little variability in the outcomes is explained by the predictors, the data is one-sided and the prediction strength is strong, as evidenced by the low RMSE. AI-RAD-diagnosed nodules were unable to predict pulmonary hospitalization, a finding that correlates with the subclinical nature of early lung neoplasia and highlights the need for screening. Pack-years, current smoking status, and AI-RAD-detected nodules significantly predicted the diagnosis of lung cancer at 1 year (AUC = 0.942, *R*^2^ = 0.139, RMSE = 0.188). Clinically, this correlates with the expected findings as smoking is the largest risk factor for cancer, and lung nodules represent possible precancerous lesions.

### Analysis of false-positive lung nodules

Our study had 56 nodules identified as false positives. Age was found to be a weak predictor of having a false-positive nodule detected by AI (AUC = 0.666, McFadden *R*^2^ = 0.06, Pr > chi (age) = 0.01, log-likelihood test = 0.007). There is roughly a 25% chance of a false-positive nodule at age 64. Furthermore, the mean age in this cohort is 68 years for those with nodules and 64 years for those without nodules, suggesting that the average patient screened in our population has a 25% chance of having a false positive consistent with prior literature [35].

Most false positives were identified as atelectasis, extrapleural fat, infection, and protruding osteophytes from thoracic vertebral bodies. Nine false positives were uncategorizable by the panel of radiologists. Atelectasis and infection were commonly misidentified as nodules likely due to their relative mass-like area of hyperdensity with adjacent normal or emphysematous lung parenchyma. The lobular contour of the extrapleural fat and protruding osteophytes from thoracic vertebral bodies, in direct contact with the lung parenchyma, likely led to their misidentification as nodules. While future study is necessary to compensate for the presence of these coincident findings, quantification of rates of known false positives may be useful when using the AI software as an adjunct tool for diagnosis.

### Limitations

Although the AI-RAD platform has been previously tested and trained on thousands of manually segmented and curated chest CT scans and validated against separate CT datasets, application to our single-center study only reflects the population findings at our institution with a small number of patients and is not yet generalizable to larger populations. Findings should be treated as a proof-of-concept in nature for the dual implementation of neural networks requiring larger multi-center validations needed to produce generalizable results.

This study is also underpowered to predict lung cancer outcomes analysis with only 1 year of follow-up. While lung nodules were a significant variable in the lung cancer multivariate model, the exact predictive contribution cannot be established yet. However, it should be noted that multiple large prospective studies have validated that lung nodules identified on LDCT are predictive of lung cancer [3–5]. AI-RAD performed admirably by collecting all nodules present in the LDCT scans (sensitivity = 1), so while multi-year follow-up is needed to readily quantify the predictive power of AI-RAD detected lung nodules, all pre-cancerous lesions were identified in this cohort. Similarly, a major limitation is the rate of false-positive nodules which was higher than expert radiologist analysis. False-positive nodules induce a significant burden on the patient in the way of unnecessary biopsies and downstream testing. More study is needed to be able to refine the parameters and provide a more specific diagnosis.

Finally, the results of this study describe the predictive power of AI-CACV and AI-LN on outcomes in separate categories. Additional investigation of the data is needed to evaluate the potential combined morbidity, mortality, and cost-benefit of AI-RAD when applied to LDCT populations. Importantly, this study has not been validated by an independent cohort. Future study with longer-term follow-up data and a larger cohort are needed for this assessment and is currently being collected.

## Conclusion

Overall, this study demonstrated a proof-of-concept model using two parallel neural networks to diagnose major contributors to mortality in a high-risk population within an existing screening framework. Results from the AI software strongly agree with expert radiologist determination of both CACV and lung nodule detection. Diagnosis of lung nodules on a per-nodule basis is highly sensitive, but poorly specific, with false-positive rates similar to expert thoracic radiologists. Major contributors to false positives include age, positing senescent changes in the lung as a confounder, and object of further study.

AI-RAD-detected CACV and lung nodules function to predict major cardiopulmonary outcomes at 1 year with excellent predictive power, giving evidence that AI measurements correspond well with their expert-read counterparts. However, the cohort was small and not generalizable to the general population. Additionally, major insights into the feasibility of AI-based CACV quantification in LDCT are presented. To our knowledge, this is the first study to evaluate the performance and predictive power of two separate AI neural networks in an already validated screening modality. Further studies are needed to expand the scope anatomically to maximize risk assessment and reduce health care costs, avoid false-positive lung nodules, and gain longer-term follow-up with major cardiopulmonary outcomes.

## Supplementary Information

**Additional file 1: Table S1.** Demographics of patients with and without lung nodules stratified by the AI and expert as well as expert CAC scores. **Table S2.** Comparison of risk factors and clinical attributes between patients with expert determined nodules, comparison of risk factors and clinical attributes between patients with AI determined nodules, and comparison of risk factors and clinical attributes between patients with CAC > 0 and CAC = 0. **Table S3.** Demographics and risk factors associated with pulmonary outcomes. **Table S4.** Demographics and risk factors associated with cardiac outcomes. **Table S5**. Simple logistic regression for parallel analysis of AI-volume and expert-volume for prediction of cardiac outcomes. **Table S6.** AUC and McFadden R^2^ for outcomes with and without AI components included in the model. **Table S7.** Summary statistics of Patients with False Positive Nodules. **Figure S1.** ROC curves for comparison of CAC AI-Volume and Expert-Volume for prediction of MACE. Expert and AI-Volume both excellently predict MACE. **Figure S2.** ROC Curves for comparison of CAC AI-Volume and Expert Volume for prediction of ACS/MI hospitalization in our study timeframe. **Figure S3.** ROC Curves for comparison of CAC AI-Volume and Expert Volume for prediction of percutaneous coronary intervention (coronary catheterization or stent placement) or coronary artery bypass graft operation. **Figure S4.** Root cause analysis of false-positive nodules. **A.** Logistic regression of having one false positive nodule as predicted by age. **B.** Logistic regression probability curve of false positive nodules as a function of age. **C.** True anatomic identities and relative frequencies of false positive nodule etiologies.

## Data Availability

MUSC used a Siemens prototype of the software, which was delivered to MUSC under a contract and Master Research Agreement and was only for use at MUSC for a limited time. Unfortunately, the algorithm cannot be shared publicly. The raw image dataset generated or analyzed during this study is not publicly available due to the DICOM metadata containing information that could compromise patient privacy/consent.

## Abbreviations

ACS/MI: Acute coronary syndrome/myocardial infarction
AI: Artificial intelligence
AUC: Area under the curve
BMI: Body mass index
BSA: Body surface area
CACV: Coronary artery calcium volume
CAD: Coronary artery disease
CI: Confidence interval
CT: Computed tomography
FN: False negative
FP: False positive
HLD: Hyperlipidemia
HTN: Hypertension
LDCT: Low-dose computed tomography
LN: Lung nodule(s)
MACE: Major adverse cardiac event
MALI: Major adverse lung incident
NPV: Negative predictive value
PCI: Percutaneous surgical intervention
PLS: Partial least squares (regression)
PPV: Positive predictive value
RMSE: Root mean square error
ROC: Receiver operator characteristic (curve)
TN: True negative
TP: True positive
VIP: Variable importance projection
