# Supplementary material for: Automated detection of lung nodules and coronary artery calcium using artificial intelligence on low-dose CT scans for lung cancer screening: accuracy and prognostic value
Source: BMC Med. 2021 Mar 4;19:55. doi: 10.1186/s12916-021-01928-3 (PMC7931546; doi:10.1186/s12916-021-01928-3)
Supplement: Supplementary file 1 — Additional file 1: Table S1. Demographics of patients with and without lung nodules stratified by the AI and expert as well as expert CAC scores. Table S2. Comparison of risk factors and clinical attributes between patients with expert determined nodules, comparison of risk factors and clinical attributes between patients with AI determined nodules, and comparison of risk factors and clinical attributes between patients with CAC > 0 and CAC = 0. Table S3. Demographics and risk factors associated with pulmonary outcomes. Table S4. Demographics and risk factors associated with cardiac outcomes. Table S5. Simple logistic regression for parallel analysis of AI-volume and expert-volume for prediction of cardiac outcomes. Table S6. AUC and McFadden R2 for outcomes with and without AI components included in the model. Table S7. Summary statistics of Patients with False Positive Nodules. Figure S1. ROC curves for comparison of CAC AI-Volume and Expert-Volume for prediction of MACE. Expert and AI-Volume both excellently predict MACE. Figure S2. ROC Curves for comparison of CAC AI-Volume and Expert Volume for prediction of ACS/MI hospitalization in our study timeframe. Figure S3. ROC Curves for comparison of CAC AI-Volume and Expert Volume for prediction of percutaneous coronary intervention (coronary catheterization or stent placement) or coronary artery bypass graft operation. Figure S4. Root cause analysis of false-positive nodules. A. Logistic regression of having one false positive nodule as predicted by age. B. Logistic regression probability curve of false positive nodules as a function of age. C. True anatomic identities and relative frequencies of false positive nodule etiologies. [file 12916_2021_1928_MOESM1_ESM.docx]

**Supplemental information**

**Automated detection of lung nodules and coronary artery calcium using artificial intelligence on low-dose CT scans for lung cancer screening: Accuracy and prognostic value**

Jordan Chamberlin BS^1^, Madison R. Kocher MD^1^, Jeffrey Waltz MD^1^, Madalyn Snoddy BS, BA^1^, Natalie F C Stringer BS^1^, Joseph Stephenson BS^1^, Pooyan Sahbaee PhD^2^, Puneet Sharma PhD^2^, Saikiran Rapaka PhD^2^, U Joseph Schoepf MD^1^, Andres F. Abadia^1^, Jonathan Sperl^2^, Phillip Hoelzer^2^, Megan Mercer MD^1^, Nayana Somayaji^1^, Gilberto Aquino MD^1^, and Jeremy R. Burt MD^1†^

^1^Department of Radiology, Medical University of South Carolina. Charleston, SC 29403

^2^ Siemens Healthineers/Princeton, NJ

^†^Corresponding author:

Jeremy R Burt, MD

MUSC-ART

25 Courtenay Drive, MSC 226

2^nd^ Floor, Rm 2256

Charleston, SC 29425

burtje@musc.edu

**Demographics**

| N = 117 | Lung nodules – AI (n = 83) | | No Lung nodules – AI (n = 34) | |
| --- | --- | --- | --- | --- |
|  | **Mean** | **SD** | **Mean** | **SD** |
| Age | 68.0 | 5.68 | 63.9 | 4.83 |
| BMI | 27.8 | 4.29 | 27.1 | 5.20 |
| BSA | 1.94 | 0.24 | 1.86 | 0.24 |
|  | **Median** | **IQR** | **Median** | **IQR** |
| Pack years | 41.0 | 33.7 – 54.5 | 35.0 | 30.0 – 40.0 |
|  | **Count** | **Frequency** | **Count** | **Frequency** |
| Male Sex | 43 | 51.8 | 13 | 38.3 |
| Caucasian | 60 | 72.3 | 18 | 52.9 |
| Hypertension | 62 | 74.7 | 25 | 73.5 |
| Hyperlipidemia | 63 | 75.9 | 18 | 52.9 |
| Diabetes | 23 | 27.1 | 11 | 32.4 |
| Current smoker | 32 | 38.6 | 20 | 60.6 |
| N = 117 | **Lung nodules – Expert (n = 69)** | | **No Lung nodules – AI (n = 48)** | |
|  | **Mean** | **SD** | **Mean** | **SD** |
| Age | 68.4 | 5.47 | 64.5 | 5.36 |
| BMI | 27.9 | 4.30 | 27.2 | 4.92 |
| BSA | 1.94 | 0.24 | 1.89 | 0.25 |
|  | **Median** | **IQR** | **Median** | **IQR** |
| Pack years | 42.0 | 33.8 – 56.0 | 35 | 30.0 – 42.0 |
|  | **Count** | **Frequency** | **Count** | **Frequency** |
| Male Sex | 34 | 49.3 | 22 | 45.8 |
| Caucasian | 49 | 71.0 | 29 | 60.4 |
| Hypertension | 50 | 72.5 | 37 | 77.1 |
| Hyperlipidemia | 52 | 75.4 | 29 | 60.4 |
| Diabetes | 18 | 26.1 | 16 | 33.3 |
| Current smoker | 26 | 37.7 | 26 | 55.4 |
|  | **Lung nodules – Expert (n = 69)** | | **Lung nodules – AI (n = 83)** | |
|  | **Mean** | **SD** | **Mean** | **SD** |
| Age | 68.4 | 5.47 | 68.0 | 5.68 |
| BMI | 27.9 | 4.30 | 27.8 | 4.29 |
| BSA | 1.94 | 0.24 | 1.94 | 0.24 |
|  | **Median** | **Median** | **Median** | **IQR** |
| Pack years | 42.0 | 33.8 – 56.0 | 41.0 | 33.7 – 54.5 |
|  | **Count** | **Frequency** | **Count** | **Frequency** |
| Male Sex | 34 | 49.3 | 43 | 51.8 |
| Caucasian | 49 | 71.0 | 60 | 72.3 |
| Hypertension | 50 | 72.5 | 62 | 74.7 |
| Hyperlipidemia | 52 | 75.4 | 63 | 75.9 |
| Diabetes | 18 | 26.1 | 23 | 27.1 |
| Current smoker | 26 | 37.7 | 32 | 38.6 |
| N = 117 | **Expert CAC Score > 0 (n = 86)** | | **Expert CAC Score = 0 (n = 31)** | |
|  | **Mean** | **SD** | **Mean** | **SD** |
| Age | 67.7 | 5.72 | 64.2 | 5.03 |
| BMI | 27.3 | 4.54 | 28.3 | 4.60 |
| BSA | 1.92 | 0.25 | 1.92 | 0.23 |
|  | **Median** | **IQR** | **Median** | **IQR** |
| Pack years | 41 | 30.0 – 56.0 | 36 | 30.9 – 38.4 |
|  | **Count** | **Frequency** | **Count** | **Frequency** |
| Male Sex | 46 | 53.5 | 10 | 32.3 |
| Caucasian | 64 | 74.4 | 14 | 45.2 |
| Hypertension | 64 | 74.4 | 23 | 74.2 |
| Hyperlipidemia | 63 | 73.3 | 18 | 58.1 |
| Diabetes | 28 | 32.6 | 6 | 19.4 |
| Current smoker | 35 | 41.2 | 17 | 54.4 |

**Table S1.** Demographics of patients with and without lung nodules stratified by the AI and expert as well as expert CAC scores. Mean + SD were reported for continuous variables determined to follow a normal distribution by the shapiro-wilks test. Variables following a non-normal distribution were reported using the median + IQR. Categorical variables were reported with count and frequency.

**Risk Factors/Clinical Attributes**

| N = 117 | Nodules – Expert (n = 69) | | No Nodules – Expert (n = 48) | | P* |
| --- | --- | --- | --- | --- | --- |
|  | **Count** | **Frequency** | **Count** | **Frequency** |  |
| TB Exposure | 3 | 4.35 | 2 | 4.17 | 1.000 |
| Asbestos Exposure | 6 | 8.70 | 3 | 6.25 | 0.735 |
| FH of Lung Cancer | 15 | 21.8 | 5 | 10.4 | 0.137 |
| COPD | 24 | 34.8 | 12 | 25.0 | 0.311 |
| ILD | 1 | 1.45 | 2 | 4.17 | 0.567 |
| H/o cardiac disease | 23 | 33.3 | 11 | 22.9 | 0.301 |
| FH of cardiac disease | 23 | 33.3 | 20 | 41.7 | 0.436 |
| Stroke/TIA h/o | 7 | 10.1 | 5 | 10.4 | 1.000 |
| Daily Aspirin | 29 | 42.0 | 19 | 39.6 | 0.850 |
| N = 117 | **Nodules – AI (n = 83)** | | **No Nodules – AI (n = 34)** | | **P*** |
|  | **Count** | **Frequency** | **Count** | **Frequency** |  |
| TB Exposure | 3 | 3.61 | 2 | 5.89 | 0.627 |
| Asbestos Exposure | 9 | 10.8 | 0 | 0 | 0.057 |
| FH of Lung Cancer | 17 | 20.5 | 3 | 8.82 | 0.178 |
| COPD | 28 | 33.7 | 8 | 23.5 | 0.378 |
| ILD | 3 | 3.61 | 0 | 0 | 0.555 |
| H/o cardiac disease | 25 | 30.1 | 9 | 26.5 | 0.824 |
| FH of cardiac disease | 26 | 31.3 | 17 | 50 | 0.090 |
| Stroke/TIA h/o | 7 | 8.43 | 5 | 14.7 | 0.327 |
| Daily Aspirin | 36 | 43.4 | 12 | 34.3 | 0.535 |
| N = 117 | **Nodules – Expert (n = 69)** | | **Nodules – AI (n = 83)** | | *** |
|  | **Count** | **Frequency** | **Count** | **Frequency** | --- |
| TB Exposure | 3 | 4.35 | 3 | 3.61 | --- |
| Asbestos Exposure | 6 | 8.70 | 9 | 10.8 | --- |
| FH of Lung Cancer | 15 | 21.8 | 17 | 20.5 | --- |
| COPD | 24 | 34.8 | 28 | 33.7 | --- |
| ILD | 1 | 1.45 | 3 | 3.61 | --- |
| H/o cardiac disease | 23 | 33.3 | 25 | 30.1 | --- |
| FH of cardiac disease | 23 | 33.3 | 26 | 31.3 | --- |
| Stroke/TIA h/o | 7 | 10.1 | 7 | 8.43 | --- |
| Daily Aspirin | 29 | 42.0 | 36 | 43.4 | --- |
| N = 114** | **AI CAC Score > 0 (n = 84)** | | **AI CAC Score = 0 (n = 30)** | | **P*** |
|  | **Count** | **Frequency** | **Count** | **Frequency** |  |
| TB Exposure | 3 | 3.57 | 2 | 6.67 | 0.606 |
| Asbestos Exposure | 8 | 9.52 | 1 | 3.33 | 0.441 |
| FH of Lung Cancer | 14 | 16.7 | 6 | 20.0 | 0.780 |
| COPD | 31 | 36.9 | 5 | 16.7 | **0.043** |
| ILD | 3 | 3.57 | 0 | 0 | 0.565 |
| H/o cardiac disease | 28 | 33.3 | 3 | 10.0 | **0.016** |
| FH of cardiac disease | 30 | 35.7 | 12 | 40.0 | 0.667 |
| Stroke/TIA h/o | 11 | 13.1 | 1 | 3.33 | 0.179 |
| Daily Aspirin | 35 | 41.7 | 10 | 33.3 | 0.516 |

**Table S2.** Comparison of risk factors and clinical attributes between patients with expert determined nodules, comparison of risk factors and clinical attributes between patients with AI determined nodules, and comparison of risk factors and clinical attributes between patients with CAC > 0 and CAC = 0.

*Comparison of between-group frequencies calculated using Fisher’s Exact Test for categorical variables. Purpose of comparison is to assess for confounding variables for use in logistic regression. α = 0.05.

** 3 Patients were excluded because of missing information.

*** Univariate statistics were not performed comparing patients with true nodules determined by expert and AI. The difference in these two populations is by definition the presence of false positives which is examined further in table S6.

| N = 117 | Pulmonary Hospitalization (n = 27) | | | Lung Cancer diagnosis (n = 5) | | | MALI** (n = 28) | | |
| --- | --- | --- | --- | --- | --- | --- | --- | --- | --- |
|  | **Mean** | |  | **Mean** | |  | **Mean** | |  |
| Demographics | **Yes** | **No** | **P** | **Yes** | **No** | **P** | **Yes** | **No** | **P** |
| Age | 67.4 | 66.6 | 0.482 | 68.6 | 66.7 | 0.466 | 67.0 | 66.7 | 0.773 |
| BMI | 27.4 | 27.6 | 0.836 | 29.3 | 27.5 | 0.403 | 27.5 | 25.6 | 0.893 |
| BSA | 1.883 | 1.929 | 0.397 | 1.92 | 1.92 | 0.957 | 1.88 | 1.93 | 0.337 |
| Pack Years* (Median) | 45 | 40 | 0.075 | 67.5 | 40.0 | **0.018** | 45 | 39 | **0.038** |
|  | **Frequency** | |  | **Frequency** | |  | **Frequency** | |  |
| Demographics | **Yes** | **No** | **P** | **Yes** | **No** | **P** | **Yes** | **No** | **P** |
| Male Sex | 0.47 | 0.48 | 1 | 0.4 | 0.48 | 1 | 0.48 | 0.46 | 1 |
| Caucasian | 0.78 | 0.64 | 0.141 | 0.8 | 0.66 | **0.029** | 0.75 | 0.65 | 0.241 |
| HTN | 0.67 | 0.78 | 0.311 | 0.8 | 0.75 | 1 | 0.68 | 0.77 | 0.326 |
| HLD | 0.70 | 0.70 | 1 | 0.8 | 0.87 | 1 | 0.68 | 0.70 | 0.816 |
| Diabetes | 0.22 | 0.31 | 0.471 | 0.8 | 0.70 | 1 | 0.20 | 0.32 | 0.348 |
| Current Smoker | 0.42 | 0.49 | 0.081 | 0 | 0.47 | 0.064 | 0.32 | 0.48 | 0.133 |
|  | **Frequency** | |  | **Frequency** | |  | **Frequency** | |  |
| Risk Factors | **Yes** | **No** | **P** | **Yes** | **No** | **P** | **Yes** | **No** | **P** |
| TB Exposure | 0.04 | 0.04 | 1 | 0 | 0.04 | 1 | 0.04 | 0.04 | 1 |
| Asbestos Exposure | 0.04 | 0.09 | 0.682 | 0 | 0.08 | 1 | 0.04 | 0.10 | 0.685 |
| FH Lung Cancer | 0.15 | 0.22 | 1 | 0.4 | 0.16 | 0.202 | 0.14 | 0.18 | 0.779 |
| COPD | 0.41 | 0.28 | 0.237 | 0 | 0.32 | 0.322 | 0.39 | 0.28 | 0.348 |
| ILD | 0 | 0.03 | 1 | 0 | 0.03 | 1 | 0 | 0.03 | 1 |
| FH Cardiac Disease | 0.33 | 0.38 | 0.821 | 0.2 | 0.38 | 0.651 | 0.32 | 0.38 | 0.656 |
| Stroke/TIA History | 0.13 | 0.10 | 1 | 0 | 0.11 | 1 | 0.17 | 0.09 | 0.478 |
| Daily Aspirin | 0.41 | 0.41 | 1 | 0.4 | 0.41 | 1 | 0.39 | 0.41 | 1 |
|  | **Mean** | |  | **Mean** | |  | **Mean** | |  |
|  | **Yes** | **No** | **P** | **Yes** | **No** | **P** | **Yes** | **No** | **P** |
| CAC Score – AI | 439 | 462 | 0.891 | 704 | 447 | 0.487 | 470 | 453 | 0.917 |
| CAC Score – Expert | 379 | 644 | 0.345 | 545 | 483 | 0.949 | 400 | 641 | 0.384 |
|  | **Frequency** | |  | **Frequency** | |  | **Frequency** | |  |
|  | **Yes** | **No** | **P** | **Yes** | **No** | **P** | **Yes** | **No** | **P** |
| Lung Nodules – AI | 0.67 | 0.72 | 0.632 | 1 | 0.70 | 0.319 | 0.65 | 0.73 | 0.474 |
| Lung Nodules – Expert | 0.67 | 0.57 | 0.382 | 1 | 0.57 | 0.077 | 0.65 | 0.57 | 0.660 |

**Table S3.** Demographics and risk factors associated with pulmonary outcomes. Continuous variables were assessed using two-tailed T-Tests and categorical variables with Fisher’s Exact Test.

*Pack years was determined to follow a non-normal distribution and was assessed using the Mann-Whitney U-Test.

**MALI (major adverse lung incident) – Comprised of pulmonary hospitalization for any cause, lung cancer diagnosis, biopsy, or lung surgery.

| N = 96** | ACS/MI hospitalization (n = 11) | | | PCI/Surgical Intervention (n = 11) | | | MACE (n = 13) | | |
| --- | --- | --- | --- | --- | --- | --- | --- | --- | --- |
|  | **Mean** | |  | **Mean** | |  | **Mean** | |  |
| Demographics | **Yes** | **No** | **P** | **Yes** | **No** | **P** | **Yes** | **No** | **P** |
| Age | 69.7 | 66.4 | 0.075 | 69.2 | 66.5 | 0.148 | 68.8 | 66.5 | 0.189 |
| BMI | 28.2 | 27.8 | 0.576 | 28.5 | 27.3 | 0.409 | 28.4 | 27.3 | 0.416 |
| BSA | 2.00 | 1.90 | 0.199 | 2.00 | 1.90 | 0.194 | 1.99 | 1.90 | 0.190 |
| Pack Years* (Median) | 45 | 40 | 0.402 | 45 | 40 | 0.087 | 45 | 40 | 0.243 |
|  | **Frequency** | |  | **Frequency** | |  | **Frequency** | |  |
| Demographics | **Yes** | **No** | **P** | **Yes** | **No** | **P** | **Yes** | **No** | **P** |
| Male Sex | 54.5 | 47.1 | 0.780 | 45.5 | 48.2 | 1.000 | 53.8 | 47.0 | 0.798 |
| Caucasian | 63.6 | 71.8 | 0.600 | 63.6 | 71.8 | 0.600 | 61.6 | 72.3 | 0.507 |
| HTN | 1.00 | 68.3 | **0.031** | 1.00 | 68.3 | **0.031** | 1.00 | 67.5 | **0.017** |
| HLD | 1.00 | 67.1 | **0.030** | 90.9 | 68.3 | 0.167 | 92.3 | 67.5 | 0.100 |
| Diabetes | 36.4 | 25.9 | 0.482 | 36.4 | 25.9 | 0.482 | 30.8 | 26.5 | 0.744 |
| Current Smoker | 36.4 | 48.3 | 0.534 | 45.5 | 52.9 | 1.000 | 46.2 | 47.0 | 1.000 |
|  | **Frequency** | |  | **Frequency** | |  | **Frequency** | |  |
| Risk Factors | **Yes** | **No** | **P** | **Yes** | **No** | **P** | **Yes** | **No** | **P** |
| TB Exposure | 9.09 | 4.71 | 0.463 | 9.09 | 4.71 | 0.463 | 7.69 | 4.82 | 0.525 |
| Asbestos Exposure | 18.2 | 5.88 | 0.182 | 9.09 | 7.06 | 0.586 | 15.4 | 6.02 | 0.240 |
| FH Lung Cancer | 27.3 | 16.5 | 0.405 | 18.2 | 17.6 | 1.000 | 23.1 | 16.9 | 0.696 |
| COPD | 63.4 | 29.4 | **0.038** | 63.6 | 29.4 | **0.038** | 53.8 | 30.1 | 0.117 |
| ILD | 9.09 | 1.18 | 0.217 | 0 | 2.35 | 1.000 | 7.69 | 1.21 | 0.254 |
| H/o Cardiac Disease | 90.9 | 16.5 | **<0.001** | 90.9 | 16.5 | **<0.001** | 92.3 | 14.5 | **<0.001** |
| FH Cardiac Disease | 18.2 | 37.7 | 0.318 | 81.8 | 62.4 | 0.318 | 15.4 | 38.6 | 0.129 |
| Stroke/TIA History | 27.3 | 8.24 | 0.086 | 27.3 | 8.24 | 0.086 | 43.8 | 7.23 | **0.028** |
| Daily Aspirin | 81.9 | 30.6 | **0.002** | 63.6 | 32.9 | 0.092 | 69.2 | 31.3 | **0.013** |
|  | **Mean** | |  | **Mean** | |  | **Mean** | |  |
|  | **Yes** | **No** | **P** | **Yes** | **No** | **P** | **Yes** | **No** | **P** |
| CAC Score – AI | 1168 | 321 | **<0.001** | 1063 | 407 | **0.005** | 1285 | 357 | **<0.001** |
| CAC Score – Expert | 1285 | 379 | **<0.001** | 1010 | 341 | **0.001** | 1120 | 292 | **<0.001** |

**Table S4.** Demographics and risk factors associated with cardiac outcomes. Continuous variables were assessed using two-tailed T-Tests and categorical variables with Fisher’s Exact Test.

*Pack years was determined to follow a non-normal distribution and was assessed using the Mann-Whitney U-Test.

**Patients with missing AI or expert data excluded from outcomes analysis (n = 18). 3 Patients excluded because they had a mislabeled stent or CABG on initial read (total excluded n = 21).

**Logistic Regression modelling of AI vs Expert CAC Volume assessment**

| **AI-Volume** | **P** | **OR (95% CI)** | **McFadden R^2^** | **AUC** |
| --- | --- | --- | --- | --- |
| **MACE** | **0.002** | 1.001 (1.000 – 1.002) | 0.167 | 0.854 |
| **ACS/MI Hospitalization** | **0.006** | 1.001 (1.000 – 1.002) | 0.127 | 0.825 |
| **PCI/Surgical intervention** | **0.019** | 1.001 (1.000 – 1.002) | 0.079 | 0.828 |
| **Expert-Volume** | **P** | **OR (95% CI)** | **McFadden R^2^** | **AUC** |
| **MACE** | 0.083 | 1.000 (1.000 – 1.001) | 0.049 | 0.835 |
| **ACS/MI Hospitalization** | 0.098 | 1.000 (1.000 – 1.001) | 0.043 | 0.814 |
| **PCI/Surgical Intervention** | 0.198 | 1.000 (1.000 – 1.001) | 0.022 | 0.801 |

**Table S5**. Simple logistic regression for parallel analysis of AI-volume and expert-volume for prediction of cardiac outcomes. AI measurements predict outcomes similarly to expert measurements and have superior model fits.

| Outcome | AUC with AI | AUC w/o AI | R^2^ with AI | R^2^ w/o AI |
| --- | --- | --- | --- | --- |
| ACS/MI | 0.900 | 0.810 | 0.257 | 0.112 |
| MACE | 0.911 | 0.785 | 0.301 | 0.109 |
| PCI/Surgical intervention | 0.881 | 0.816 | 0.173 | 0.104 |
| Pulmonary hospitalization | 0.734 | 0.695 | 0.142 | 0.115 |
| Lung Cancer | 0.942 | 0.941 | 0.139 | 0.155 |

**Table S6.** AUC and McFadden R^2^ for outcomes with and without AI components included in the model.

**False Positive Nodule Analysis**

|  | False Positive Nodule | | No False Positive | | P |
| --- | --- | --- | --- | --- | --- |
|  | **Mean** | **SD** | **Mean** | **SD** |  |
| Age | 68.7 | 6.14 | 65.8 | 5.27 | **0.010** |
| BMI | 27.1 | 4.25 | 28.0 | 4.60 | 0.350 |
| BSA | 1.90 | 0.27 | 1.93 | 0.22 | 0.517 |
| Pack Years | 41.8 | 23.1 | 45.5 | 24.0 | 0.424 |
|  | **Count** | **Frequency** | **Count** | **Frequency** |  |
| Male Sex | 17 | 42.5 | 37 | 48.7 | 0.710 |
| Caucasian | 11 | 27.5 | 25 | 32.9 | 0.526 |
| HTN | 30 | 75.0 | 57 | 75.0 | 1.000 |
| HLD | 29 | 72.5 | 52 | 68.4 | 0.667 |
| Diabetes | 14 | 35.0 | 20 | 26.3 | 0.392 |
| Current Smoker | 15 | 37.5 | 37 | 48.7 | 0.326 |
| TB Exposure | 1 | 2.50 | 4 | 5.26 | 0.658 |
| Asbestos Exposure | 3 | 7.50 | 6 | 7.90 | 1.000 |
| FH Lung Cancer | 6 | 15.0 | 14 | 18.4 | 0.798 |
| COPD | 11 | 27.5 | 25 | 32.9 | 0.674 |
| ILD | 1 | 2.50 | 2 | 2.63 | 1.000 |

**Table S7.** Summary statistics of Patients with False Positive Nodules. Continuous variables were assessed with Two-Tailed T-Tests and categorical variables by Fisher’s Exact Test.

**
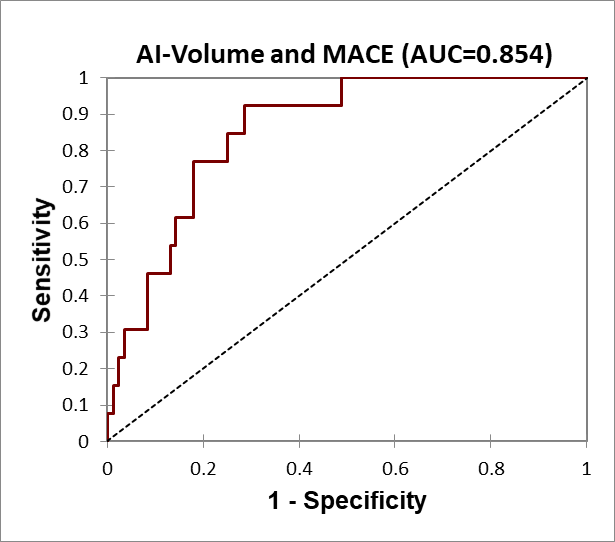

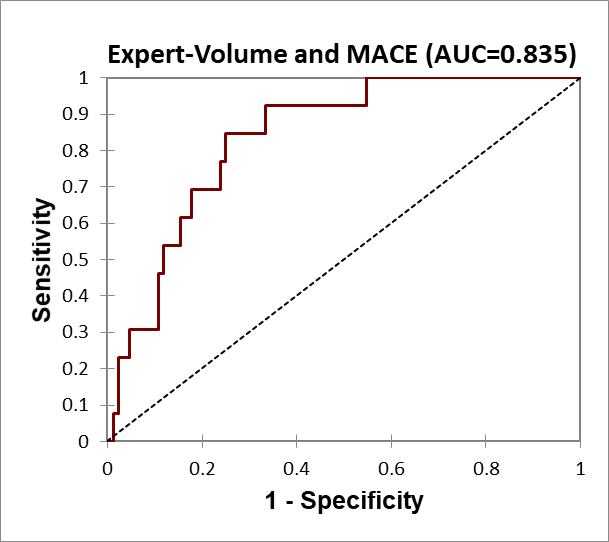
**

**Figure S1.** ROC curves for comparison of CAC AI-Volume and Expert-Volume for prediction of MACE. Expert and AI-Volume both excellently predict MACE.


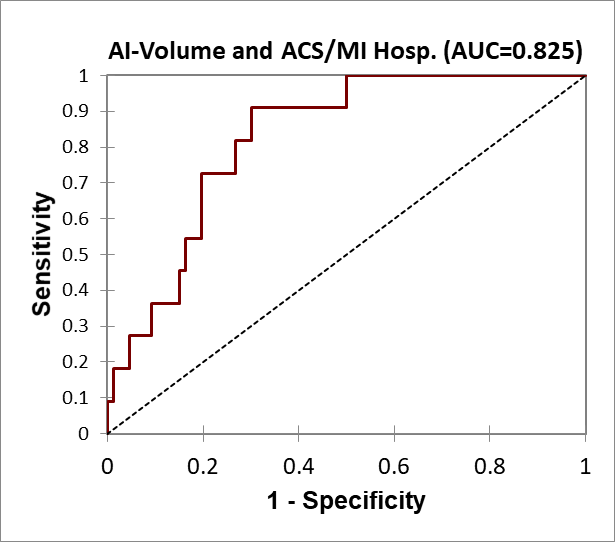

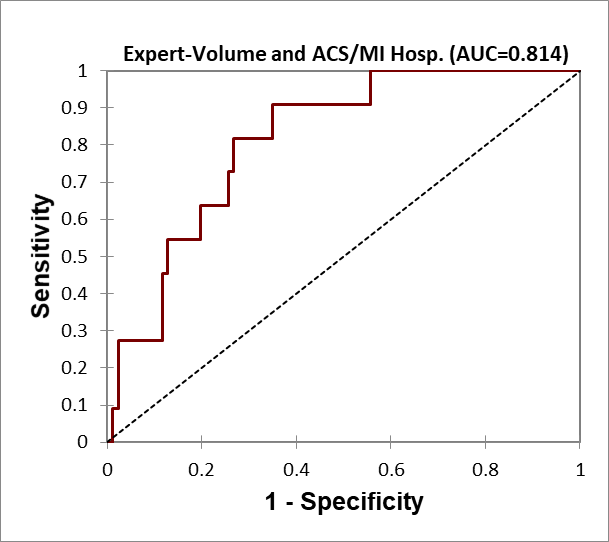


**Figure S2.** ROC Curves for comparison of CAC AI-Volume and Expert Volume for prediction of ACS/MI hospitalization in our study timeframe. Both Expert and AI volume excellently predict ACS/MI hospitalization.


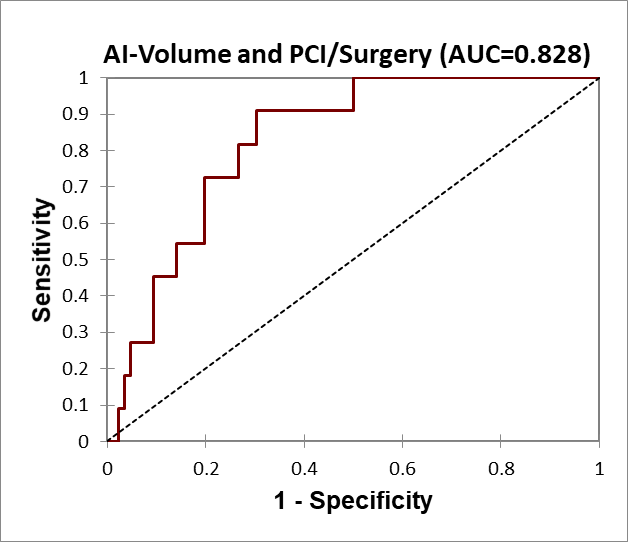

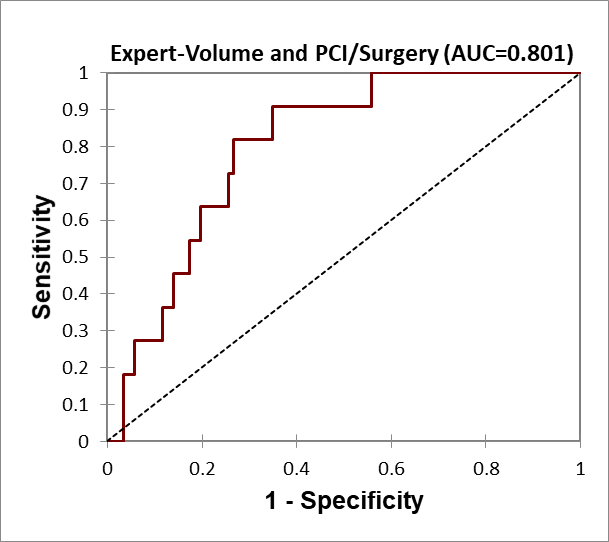


**Figure S3.** ROC Curves for comparison of CAC AI-Volume and Expert Volume for prediction of percutaneous coronary intervention (coronary catheterization or stent placement) or coronary artery bypass graft operation.


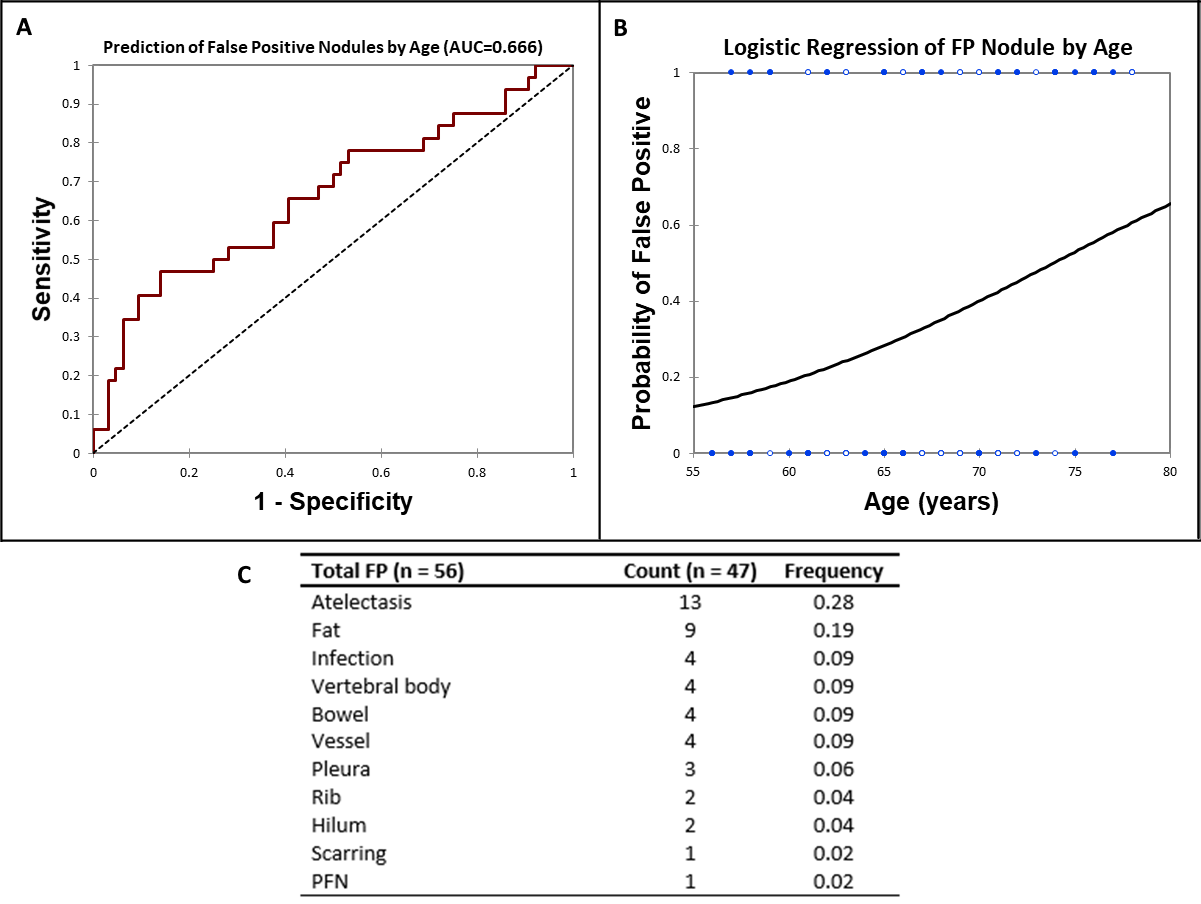


**Figure S4.** Root cause analysis of false-positive nodules. Univariate statistics of demographics and risk factors comparing patients with and without a false positive nodule and are reported in the supplemental figures. Age was significantly higher (68.7 years vs 65.8 years) in patients with false-positive nodules. **A.** Logistic regression of having one false positive nodule as predicted by age. McFadden R^2^ = 0.060, Pr > Chi (age) = 0.01, likelihood ratio test = 0.007. Age is a weak, but significant, predictor of having at least one false positive nodule (AUC = 0.666). **B.** Logistic regression probability curve of false positive nodules as a function of age. There is a 25% probability of having a false positive nodule at 64 years, 50% probability at 74 years, and 75% probability at 84 years. **C.** True anatomic identities and relative frequencies of false positive nodule etiologies. The most common false positive etiology was atelectasis (28%), followed by fat (19%), and vertebral bodies, infection, bowel, and vessels (9% respectively). 9 nodule identities were unable to be determined.
